# Selection of reliable reference genes for normalization of quantitative RT-PCR from different developmental stages and tissues in amphioxus

**DOI:** 10.1038/srep37549

**Published:** 2016-11-21

**Authors:** Qi-Lin Zhang, Qian-Hua Zhu, Xin Liao, Xiu-Qiang Wang, Tao Chen, Han-Ting Xu, Juan Wang, Ming-Long Yuan, Jun-Yuan Chen

**Affiliations:** 1 Evo-devo Institute, School of Life Sciences, Nanjing University, Nanjing 210008, China; 2 BGI-Shenzhen, Shenzhen, 518000, China; 3 State Key Laboratory of Grassland Agro-Ecosystems, College of Pastoral Agricultural Science and Technology, Lanzhou University, Lanzhou 730020, China; 4 Nanjing Institute of Geology and Paleontology, Nanjing 210008, China

## Abstract

Amphioxus is a closest living proxy to the ancestor of cephalochordates with vertebrates, and key animal for novel understanding in the evolutionary origin of vertebrate body plan, genome, tissues and immune system. Reliable analyses using quantitative real-time PCR (qRT-PCR) for answering these scientific questions is heavily dependent on reliable reference genes (RGs). In this study, we evaluated stability of thirteen candidate RGs in qRT-PCR for different developmental stages and tissues of amphioxus by four independent (geNorm, NormFinder, BestKeeper and deltaCt) and one comparative algorithms (RefFinder). The results showed that the top two stable RGs were the following: (1) *S20* and *18* *S* in thirteen developmental stages, (2) *EF1A* and *ACT* in seven normal tissues, (3) *S20* and *L13* in both intestine and hepatic caecum challenged with lipopolysaccharide (LPS), and (4) *S20* and *EF1A* in gill challenged with LPS. The expression profiles of two target genes (*EYA* and *HHEX*) in thirteen developmental stages were used to confirm the reliability of chosen RGs. This study identified optimal RGs that can be used to accurately measure gene expression under these conditions, which will benefit evolutionary and functional genomics studies in amphioxus.

Quantitative real-time PCR (qRT-PCR) technique is an important tool for exploring gene expression for a serial of target genes under different experimental conditions and has been widely used in genetic, signaling and evolutionary studies. Although semi-quantitative PCR, northern blotting and RNA sequencing could be used for detecting gene expression, qRT-PCR is considered as the most sensitive, accurate, reproductive and low-cost method for gene expression analyses^1^. However, a successful qRT-PCR strongly depends on reliable reference genes (RGs) which can correct the variations from experimental procedures (e.g. variations and quantification error between samples)^2^. Ideally, the expression levels of optimal RGs should not be influenced by different experimental conditions^3^. However, expression profiles of commonly used RGs (e.g. 18 S ribosomal RNA, 18 *S*; beta actin, *ACT*; elongation factor 1 alpha, *EF1A*; glyceraldehydes 3 phosphate dehydrogenase, *GAPDH*; alpha-tubulin, *TUB*; and ubiquitin-conjugating enzyme, *UBC*) were not universally stable among different experiments, because these RGs usually participated in mRNA translation and cell metabolism^4^,^5^,^6^. Therefore, RGs with stable expression as internal controls are essential for normalizing methods and we should identify the validated RGs for calibration purposes of different experimental conditions (e. g. developmental stages, tissues, cells and particularly treated tissues).

Living cephalochordates are broadly known as amphioxus or lancelet^7^,^8^, and it retains the most similarity of a body plan and morphology with the common ancestor (Cambrian chordates) of 550 Myr ago between cephalochordates and vertebrates. Therefore, amphioxus is a key experimental animal for studying evo-devo, evolutionary origin of organs and comparative immunology of vertebrates^7^,^8^. Amphioxus is also susceptible to antigen [e.g. stimulation of lipopolysaccharide (LPS) or bacteria], the same as vertebrates^9^. To date, whole genome have been sequenced for two amphioxus species *Branchiostoma floridae* and *B. belcheri*^8^,^10^, which provides an unprecedented opportunity to investigate origin of tissues and organs, evolution of genes related with development and immunity for vertebrates. Recently, ten tissue-specific transcriptomes of *B. belcheri* have been sequenced and our cooperators constructed online database (unpublished, http://wcy.pkusulab.com). Moreover, it has been performed successfully for artificial feeding of amphioxus^11^. To take advantage of these genomic and sample resources, establishing a standardized qRT-PCR procedure in amphioxus following the MIQE (Minimum Information for publication of Quantitative real-time PCR Experiments) guidelines will be useful for the further functional genomics studies^12^. In chordate phylum, many studies on identification of optimal RGs for gene expression normalization mainly focused on vertebrates, such as mammals, birds, fish and amphibian^13^,^14^,^15^,^16^, but information in amphioxus was limited. Several traditional RGs (*18* *S*, *ACT*, *EF1A*, *GAPDH*, *TUB* and *UBC*) have been widely used in qRT-PCR analyses in amphioxus, insects, human and plant^17^,^18^,^19^,^20^, but expression level of these RGs were variable to some degree under different conditions^2^,^21^. Wang *et al.* firstly evaluated the reliability of candidate RGs and found *EF1A* was a useful RG in different tissues of amphioxus, but number of candidate RGs and algorithms were few in the study (four candidate RGs and two statistical methods)^9^. Therefore, further research is needed to evaluate more candidate RGs under more experimental conditions with more statistical algorithms, which will improve qRT-PCR reliability and accuracy for amphioxus studies.

In this study, we aimed at identifying optimal RGs and recommending available normalization factors (NFs) for qRT-PCR analysis under different experimental conditions in amphioxus. Thirteen candidate RGs were analyzed under thirteen different developmental stages, seven different normal tissues and three challenged tissues with LPS. Besides six traditional RGs in amphioxus, the other seven candidate RGs were cyclophilin (*CYC*)^22^, glucose 6-phosphate dehydrogenase (*G6PDH*)^23^, hypoxanthine-guanine phosphoribosyltransferase (*HPRT*)^24^, ribosomal protein L3 (*L3*)^25^, ribosomal protein L13 (*L13*)^26^, ribosomal protein S20 (*S20*)^15^ and TATA box binding protein (*TBP*)^27^. Expression stability of RGs in qRT-PCR was evaluated with five algorithms (geNorm^28^, NormFinder^29^, BestKeeper^30^, deltaCt method^31^ and RefFinder^32^). Considering limits of economic, time and experimental materials in a real situation of lab, we also analyzed correlation between optimal normalization factors (NFs) from geNorm algorithm and less NFs by using Pearson correlation coefficient (*r*) for each experimental condition, and recommended the reliable NFs and the least number of RGs to satisfy experiments in reality. Two target genes, encoding eyes absent protein (*EYA*) and hematopoietically expressed homeobox (*HHEX*) which are important during embryonic development of chordates^33^,^34^, were chosen to determinate validation of selected RGs by three normalization strategies (recommended, optimal and the worst NFs) in different developmental stages of *B. belcheri*.

## Results

### Determination of primer validation and description of expression profiles for each gene

Dissociation curve analysis of each gene showed a single peak and no signal was detected in the negative controls, indicating the specificity of the primer pairs used in this study (Supplementary Fig. S1). A single band with the expected size from mixed cDNA was also detected by agarose gel electrophoresis (Supplementary Fig. S2). A standard curve was generated for each gene based on ten-fold serial dilution of the pooled cDNA (Supplementary Fig. S3). The amplification efficiency (E) values of all candidate RGs ranged from 93.87 to 103.89% with the correlation coefficient (R^2^) values varying from 0.990 to 0.999 (Table 1).

The mean value (Ctmean) and standard deviation (SD) of Ct were used to describe the levels of mRNA and variable expression of candidate RGs. The Ctmean and SD of candidate RGs in five different experimental groups were displayed in Supplementary Table S1. The results showed a wide expression range and distribution of different expression pattern for thirteen candidate RGs in different experimental groups (Supplementary Fig. S4). Except for *L3* (Ctmean = 29.02) in different developmental stages, the highest Ctmean was *HPRT* (Ctmean = 24.93–28.82) in the other four groups. Correspondingly, the lowest Ctmean was *G6PDH* (Ctmean = 16.48) in different developmental stages, whereas *18* *S* (Ctmean = 15.72–18.89) in the other four groups. Therefore, expression level of *L3* and *HPRT* was the least, while the most expressed genes were *G6PDH* and *18* *S*. Three genes (*L3*, *L13* and *S20*) with the highest SD values were the most variable in five experimental groups. Conversely, three genes (*S20*, *EF1A* and *18* *S*) showed little variation in expression levels, indicating that they were highly stable in their own experiment group (Supplementary Fig. S4 and Supplementary Table S1).

### Determination for expression stability of reference genes in different developmental stages

We calculated ranking of expression stability for thirteen candidate RGs by using four independent and a comprehensive algorithms (Table 2). The geNorm demonstrated that *18* *S* and *CYC* were the most stable genes, followed by *S20*, *G6PDH* and *TBP* (Supplementary Fig. S5), whereas the NormFinder showed that three RGs (*EF1A*, *S20* and *18* *S*) had the most expression stability. Beside, these three RGs showed the lowest intra-group variation (0.162, 0.175 and 0.183; Supplementary Table S2), which was a key factor for its ranking in NormFinder^29^. We also used the BestKeeper and deltaCt algorithms to rank the expression stability for each RG, and two genes (*S20* and *18* *S*, *EF1A* and *S20*) were the most reliable for normalization (Supplementary Table S3). The results of comprehensive ranking with the RefFinder showed that the most stable genes were *S20*, *18* *S* and *EF1A*, whereas four genes (*GAPDH*, *HPRT*, *ACT* and *L13*) were unstable (GM > 9.0).

### Determination for expression stability of reference genes in different tissues

For seven normal tissues, the three genes (*EF1A, ACT* and *G6PDH*) were the most stable by the geNorm analyses (Supplementary Fig. S5), which was consistent with the results of the deltaCt and Bestkeeper analyses. NormFinder consistently supported *TBP*, *EF1A* and *G6PDH* as the top three ranked RGs. The results of comprehensive ranking with the RefFinder showed that three genes (*EF1A*, *ACT* and *G6PDH*) were the most stable, while four genes (*18* *S*, *CYC*, *L3* and *S20*) exhibited unstable expressions (Table 3, Supplementary Tables S2 and S3).

### Determination for expression stability of reference genes in three different challenged tissues with LPS

In the intestine challenged with LPS, three genes (*S20*, *L13* and *UBC*) were the most stable analyzed by three independent algorithms (geNorm, NormFinder and deltaCt), whereas BestKeeper supported *18* *S*, *G6PDH* and *TBP* as the top three RGs. In the gill challenged with LPS, three genes (*S20*, *EF1A* and *UBC*) were the most stable in all independent analyses, except for the BestKeeper analyses where *18* *S*, *ACT* and *G6PDH* were the three top RGs. In the hepatic caecum challenged with LPS, geNorm and BestKeeper recommended *S20*, *L13* and *TBP* as the top three stable genes, whereas three most stable genes were supported by NormFinder (*S20*, *ACT* and *L13*) and deltaCt (*L13*, *ACT* and *S20*) (Tables 4, 5 and 6, Supplementary Fig. S5, Supplementary Tables S2 and S3).

The results of comprehensive ranking with the RefFinder showed that *S20* and *L13* were the most stable genes in both challenged intestine and hepatic caecum, followed by *EF1A* in the former and *TBP* in the latter. In addition to *S20*, *EF1A* and *UBC* were the most stable genes in challenged gill. *L3* was consistently recommended as the most unstable gene, followed by *CYC* in challenged intestine and gill, *18* *S*, *G6PDH* and *GAPDH* in challenged hepatic caecum.

### Determinate optimal number of normalization factors under each experimental condition

**Figure 1:**
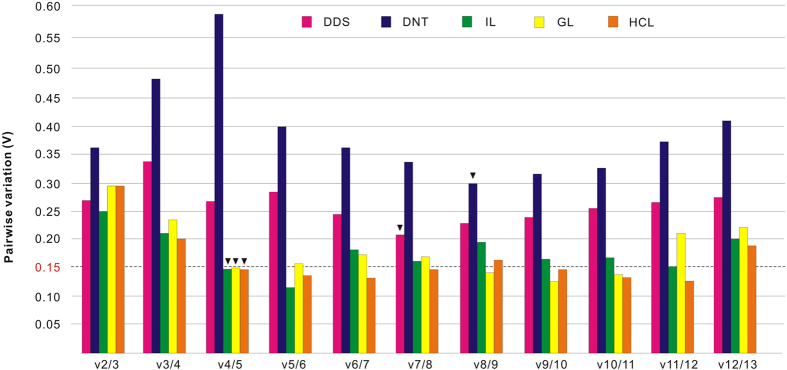
Pairwise variation (Vn/Vn + 1) analysis for selecting optimal number of reference genes in normalization of *B. belcheri* with the geNorm algorithm. DDS, different developmental stages; DNT, different normal tissues; IL, challenged intestine with LPS; GL, challenged gill with LPS; HCL, challenged hepatic caecum with LPS. The optimal number of NFs was marked by a reverse triangle with black for five groups.

To determinate optimal NFs number for normalization, the pairwise variation value (V_n_/V_n+1,_ V-value) was calculated by geNorm. If V-value is firstly lower than the default value 0.15 or lowest value in all pairwise variation^28^, the number of gene pairings will be sufficient for the consistent normalization. In different developmental stages, V7/8 showed a minimum V-value, suggesting that the optimal number of RGs for normalization was seven. V8/9 with the lowest V-value indicated that eight stable reference genes were reliable as NFs in the different normal tissues. For three challenged tissues with LPS, V-values of V4/5 were first lower than 0.15, suggesting that four RGs could be used for normalization in three tissues challenged with LPS (Fig. 1).

However, it is time-consuming and expensive for normalization by using excess RGs in the actual experiments, especially when large number of the target genes and the rare experimental samples are used. Generally, a reliable result could be obtained by using three or more RGs for normalization^28^. To further identify the reliable and the least number of NFs, we calculated the correlation between NF_n_ (n ranging from 3 to NF_opt_−1) and optimal number of NFs from geNorm (NF_opt_) for each experimental condition by Pearson correlation coefficient (*r*). Then we considered NF_n_, which contained the minimum number of RGs and no significant difference with NF_opt_, as a target number of NFs. The results showed high correlation (*r* > 0.8) between the NF_5_ and NF_opt_ (*r* = 0.83, *p* < 0.01), indicating that normalization for expression level of target genes by combining the five most stable RGs (NF_5_) could obtain the same reliable results as normalization of NF_opt_ in different normal tissues. The same is true for the normalization of the target genes in different developmental stages (*r* = 0.85, *p* < 0.01), challenged intestine (*r* = 0.99, *p* < 0.01) and gill (*r* = 0.97, *p* < 0.01) using top three stable RGs (NF_3_). However, four top RGs (NF_4=opt_) may be necessary for normalization in challenged hepatic caecum due to low correlation between NF_3_ and NF_opt_ though it reached the significant level (*r* = 0.69, *p* = 0.046) (Supplementary Fig. S6).

### Determination of reference gene validation in different developmental stages of B. belcheri

**Figure 2:**
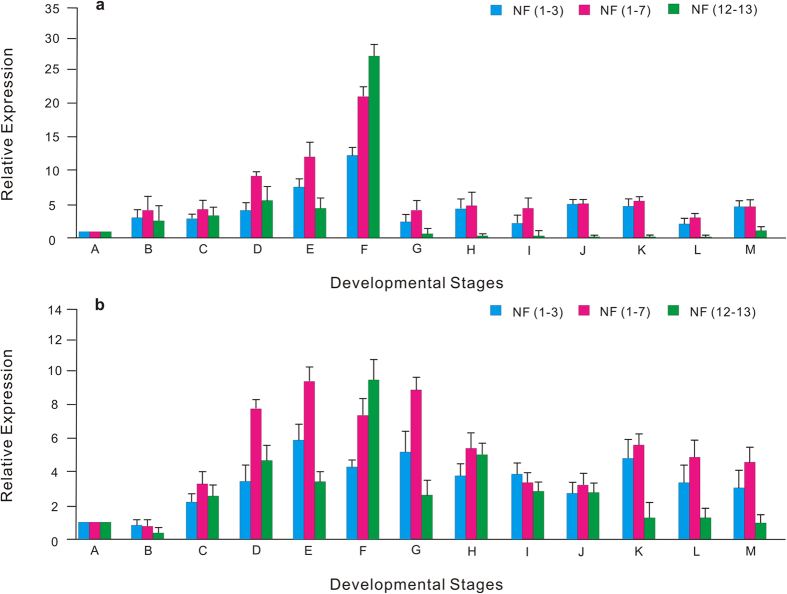
Expression profiles of the *EYA* (**a**) and *HHEX* (**b**) based on different normalization factors under different developmental stages. A, 8-cell stage; B, morula stage; C, blastula stage; D, middle gastrula stage; E, late gastrula stage; F, neurula stage; G, hatching stage; H, 10-somites stage; I, mouse-opening stage; J, 2-gill arch stage; K, two weeks after fertilization; L, two month after fertilization; M, adult stage. The normalization of *EYA* were performed by using the top three [NF (1–3)], the top seven reference genes [NF (1–7)], and two reference gene with the least stability (NF 12–13). Data were exhibited as mean ± standard deviation.

We performed expression profile analyses for two target genes (*EYA* and *HHEX*) using three [*S20*, *18* *S* and *EF1A*; NF (1–3)], seven of the most stable genes [*S20*, *18* *S*, *EF1A*, *CYC*, *G6PDH*, *UBC* and *L3*; NF (1–7)] as well as two of the least stable RGs [*ACT* and *L13*; NF (12–13)] as NFs for the normalization (Fig. 2 and Supplementary Fig. S7).

Comparing with middle gastrulae, a down-regulation of *EYA* expression was observed during later gastrulae stage by using NF (12–13) for normalization. Instead, when we used NF (1–3) and NF (1–7), we observed an up-regulation of the *EYA* expression. From hatching stage to adult stage of amphioxus, the highest expression level of *EYA* was observed during adult stage normalized with NF (12–13), but 2-gill arch stage and two week after fertilization showed the highest expression level with NF (1–3) or NF (1–7) for normalization. The highest expression level of *EYA* (neurula stage) relative to lowest one (two month after fertilization) reached 694-fold when using NF (12–13) for normalization. However, using NF (1–3) or NF (1–7) for normalization of *EYA*, the highest expression level (neurula stage) relative to lowest one (8-cell stage) was 8- and 20-fold, respectively. These results showed that high expression of *EYA* mainly happened during embryonic stages. Expression level of *EYA* reached to peak of transcripts abundant at neurul stage, and maintained the lowest expression level at 8-cell stage. Further, expression of *EYA* still kept the higher level from hatching to adult than 8-cell stage, indicating *EYA* had an important role in developmental process after embryonic stages. *HHEX* had a maximum expression level at later gastrula stage when using NF (1–3) or NF (1–7) for normalization, but it showed the highest expression level at neurula stage by normalization of NF (12–13). Although we investigated the down-regulation of these two target genes after neurula or hatching, medium expression levels were found after embryonic stages with slight up-regulation at later developmental stages. However, the expression profiles of *EYA* and *HHEX* evidently were altered and distorted when using NF (12–13) for normalization.

## Discussion

Expression patterns of target genes by qRT-PCR analysis in amphioxus are important for exploring development homology, gene evolution and comparative immunology between vertebrates and cephalochordates. According to MIQE guidelines, the use of RGs as internal controls is the most appropriate normalization way for qRT-PCR analyses^12^. In this study, we firstly performed analyses for reliability evaluation of candidate RGs in different developmental stages (especially for embryonic stages), challenged intestine, gill and hepatic caecum with LPS, as well as further determined and improved reliable RGs in different normal tissues of amphioxus.

Comparative analyses showed a high consistency for the ranking of stability for thirteen candidate RGs under each experimental condition among different statistical methods. For example, the results from the deltaCt method were highly similar to that of geNorm and NormFinder calculations, because 2/3 or even all the three top stable RGs were consistent among these three statistical algorithms. However, there were also substantial discrepancies among the results from different algorithms for each experimental group, due to different statistical models in each algorithm, as found in other studies^35^,^36^. Compared to the other three independent algorithms, the BestKeeper exhibited the most discrepancies, as has been reported in previous studies^37^. In order to overcome differences among different algorithms, we performed overall ranking for thirteen candidates RGs based on the fifth algorithm (RefFinder) to obtain the final stability. In the different developmental stages, the three RGs (*S20*, *18* *S* and *EF1A*) were identified as the most reliable, while the other three RGs (*L13*, *HPRT* and *ACT*) should be avoided in future study due to the exhibition of their low stability in all algorithms. In the different normal tissues of amphioxus, *EF1A* exhibited the most stability, consisting with the previous studies^9^. In contrast, the four RGs (*S20*, *L3*, *CYC* and *18* *S*) should not be considered for normalization of tissue-specific gene expressions. Among all candidate RGs, *S20* was ranked as a universal RG within three different tissues challenged with LPS, while *L3* was one with the least stability. Besides, stability ranking of candidate RGs with GM value < 9 in three challenged tissues by LPS was also highly similar, suggesting that selecting the same RGs was reliable for normalization in homologous organs of amphioxus under immune-stimulation conditions.

*S20* was a reliable RG in different tissues and immune relevant tissues of Atlantic salmon (*Salmo salar*)^15^, in different *Sesamia inferens* tissues and fifth instars larva treated by different temperatures^38^, but this gene exhibited the least stable expression by geNorm analysis in fruitfly by injury treatments^39^. *EF1A* was a stable RG in different developmental stages of *Sesamia inferens* and intestinal tissues of sea bass (*Dicentrarchus labrax*)^38^,^40^, but was the most variable gene in virus-infected plant hoppers^41^. In our study, no matter in different developmental stages, different tissues or three challenged tissues of amphioxus, *S20* and *EF1A* had a good performance for their expression stability, which was very similar to that of Atlantic salmon^15^, but was discrepant with virus-insects pathosystems^41^. The three genes (*18* *S*, *ACT* and *GAPDH*) were not a good choice as RGs in developmental stages of *Monopterus albus*^42^, but were considered as reliable ones in virus-immune cell^18^ and human prostate cancer^14^. Our results showed that only in different developmental stages *18* *S* was a reliable RG, whereas *ACT* and *GAPDH* exhibited the higher variations in different experimental conditions. These variations indicated that expression stability was different for the same RGs among multiple species and different RGs in the same experimental conditions, so the determination of RGs validation was essential for each specific condition in further experiments. *L13* was not considered as a stable RG in avian species^13^; however, in our study stability ranking of this gene reached top three in intestine and hepatic caecum challenged with LPS. Therefore, unconventional RGs (e.g. *L13*) should not be ignored as candidate ones in further experiments of RGs selection.

According to previous studies, use of multiple RGs for normalization could obtain more accurate results than single RG^28^, because a biased normalization can be revised by a RG combination. An optimal number of NFs for normalization was evaluated by pairwise variation analysis in the geNorm. We further calculated correlation between NF_n_ and NF_opt_ to decrease number of NFs as soon as possible under precondition of no effect on accuracy of normalization. Our results will provide a practical number of NFs in common experiments.

The validation of selected RGs was confirmed by expression profiles of two target genes (*EYA* and *HHEX*). EYA gene family comprises of four members in vertebrates (i.e. *EYA1*, *EYA2*, *EYA3* and *EYA4*) and modulates cell proliferation by phosphatase function to activate specific gene targets^43^. EYA also involves in innate immunity, DNA repair, cell migration, and cancer metastasis in adult vertebrates^44^. However, only one *EYA* was found in amphioxus and its indispensability for early development of amphioxus has been exhibited, potentially interacting with other gene functions^34^. Origin of *EYA* could be traced to fruitfly and it regulates eye development involved in cell proliferation, patterning, and neuronal information for invertebrates^45^. *HHEX* encodes an oligomeric homeodomain-containing transcription factor, and it was firstly cloned in hematopoietic tissues and highly conserved evolution in vertebrates^46^,^47^,^48^. *HHEX* is essential for embryonic development, especially for liver, thyroid and forebrain in mammal^33^. It is highly expressed in many kinds of hematopoietic cells, such as stem cells, myeloid and lymphoid progenitors^49^. Expression profiles of these two target genes showed that the transcript abundance of *EYA* and *HHEX* was strongly influenced with the development of amphioxus. Expression level of *EYA* and *HHEX* after normalization by NF (12–13) showed a huge difference from the results based on NF (1–3) or NF (1–7). Therefore, the normalization results based on NF (12–13) could not truthfully reflect the expression level of target genes in amphioxus. We found that high expression level of *HHEX* mainly concentrated at embryonic stages, which was also observed in endostyle of amphioxus by transcriptome of different amphioxus tissues (http://wcy.pkusulab.com/) and whole mount *in situ* hybridization (data not shown). Previous study reported that lymphocyte-like cells were found in endostyle of sea squirt and this tissue may be the germinal center of adult stem cells^50^, implying that endostyle played a key role in amphioxus immunity and rudiment of hematopoietic cell had been formed in cephalochordate.

Overall, we obtained two RG sets (under development and adult tissues) for normalization of target genes. The RG sets under adult tissues included two treatment types (normal and immune-stimulation): one RG subset of normal tissues was used to normalize genes of tissue-specific expression for adult amphioxus, whereas the other three RG subsets under three tissues (intestine, gill and hepatic) challenged with LPS were used to normalize immune-related gene expression for adult amphioxus. We found that there was no consensus in the RG sets to normalize data coming from adult tissues of amphioxus, and this was why we divided adult tissues into treated and untreated groups. In our present study, selection of RG sets for each of treated tissues was performed independently. We demonstrated that considerable variations of RG sets were found among adult tissues of amphioxus. These 13 candidate RGs were traditional RGs and stable RGs in other animals, so they could be potentially used for normalizing *B. belcheri* samples, particularly under development, normal tissues, challenged intestine, gill and hepatic caecum with LPS. The reliable RGs obtained here will be helpful for evolutionary and functional genomics studies in amphioxus. For other experimental conditions, however, it will be essential to evaluate the stability of 13 candidate RGs by standard process according to our manuscript.

## Methods

### Sample preparation

Adult specimens of *B. belcheri* were collected from the South China Sea (Maoming, Guangdong province, China) and reared in the cement pool with cuboid shape (1 m × 1 m × 1.2 m). We selected male and female amphioxus with a full gonad during the breeding season, and placed them into plastic cups (600 ml) with pre-filter sand and seawater in a dark place. Because most individuals were spawning during night, we collected their sperms and eggs every 30 minutes from 20:00 everyday till obtained enough experimental samples. Fertilization was performed by mixing sperms and eggs, and developmental stages were determined by using the microscope (Olympus DP71, Japan). Because of a small size for embryonic stages, we used self-made and small-bore bags consisting of stainless steel (400 mesh) to enrich experimental samples by filtering seawater that contained target stages. A total of thirteen different developmental stages were used in this study, including embryonic stages (i.e. 8-cell, morula, blastula, middle gastrula, late gastrula and neurula stages) (Supplementary Fig. S8), hatching stage, 10-somites stage, mouse-opening stage, 2-gill arch stage, two weeks after fertilization, two months after fertilization and adult stage. For each developmental stage, three biological replications were used.

Approximate 150 adult individuals were averagely put in three acrylic tanks (i.e. three biological replications) with filtered seawater, and were feed for several days to empty their contents in intestine and hepatic caecum. Seven different normal tissues (intestine, hepatic caecum, gill, skin, notochord, neural tube and muscle) were obtained from approximate 50 individuals in each tank.

We collected three tissues (intestine, gill and hepatic caecum) of *B. belcheri* which were challenged with 1 mg/ml LPS following the method of previous studies^51^,^52^. For each tissue, samples with three biological replications were collected at nine timing points of immunostimulation (0 h, 2 h, 4 h, 6 h, 12 h, 24 h, 36 h, 48 h and 60 h). Each sample contained approximate 25 adult individuals. Before immunostimulation, adult individuals with empty contents of intestine and hepatic caecum were feed in several 1.5 L tanks that were filled with 1 L sterilized seawater. The samples collected at 0 h were the controls, which were treated with PBS (it is used to dissolve LPS powder to 1 mg/ml) and then reared in seawater.

All samples were put into a 1.5 ml RNase-free microcentrifuge tube containing 1 ml of TRIzol reagent (Invitrogen, Carlsbad, CA), stored overnight at 4 °C, and transferred to −20 °C till use.

### Total RNA extraction and cDNA synthesis

Total RNA was isolated from each sample, as was previously described^53^. Residual genomic DNA was digested by RNase-free DNase Set (Qiagen, Germany) according to the manufacturer’s instructions. The RNA concentration was quantified by measuring the absorbance at 260 nm using BioPhotometer Plus (Eppendorf, Germany). Quality of the total RNA was assessed by estimating the OD_260_/_280_ with expected values between 1.8 and 2.0. RNA structural integrity was verified on agarose gel electrophoresis.

Single-stranded cDNA was synthesized using 1~5 μg of total RNA using an RervertAid First Strand cDNA Synthesis Kit (Thermo Fisher Scientific, USA) with oligo(dT)_18_ primers according to the manufacturer’s protocol. Diluted cDNA (100 ng/μl) with free-RNase water was used for further experiments.

### Identification of reference genes in the *B. belcheri* genome

We downloaded all target sequences annotated in GenBank and manually determined unannotated sequences based on the Chinese amphioxus genome (version *B.belcheri*_v18h27.r3)^10^. The coding sequence (CDS) set was downloaded from the website (http://mosas.sysu.edu.cn/) and annotated seed sequences of other model animals were hit to CDS set by using Blastn 2.2.24 (E-value 0.001). Candidate target sequences were further determined manually by using online BLAST in NCBI.

### Reference genes selection, primer design and amplification efficiency test

We selected thirteen candidate RGs to investigate their robustness as internal controls for qRT-PCR and two target genes to verify the validation of recommended RGs (Table 1). Gene-specific primers were designed by Beacon Designer 7 software. The specific amplification of all candidate/target genes was confirmed by a single band of appropriate size in a 1.5% agarose gel electrophoresis. The qRT-PCR efficiency was determined for each gene by using slope analysis with a linear regression model. A pool of all of the cDNA samples was used to calculate the PCR efficiency and correlation coefficient (*R*^2^) for each primer pair based on the standard curve method. Standard curves of five points were generated with serial dilutions of cDNA (1/10, 1/100, 1/1000, 1/10000, and 1/100000). The corresponding qRT-PCR efficiencies (E) were calculated according to the equation^54^: E (%) = (10^(−1/slope)^ − 1) × 100.

### Quantitative real-time PCR

qRT-PCR was performed using ABI 7300 real-time PCR system (Applied Biosystems, USA). cDNA was amplified in 96-well plates using the SYBR Premix Ex Taq (Takara, Japan) according to the manufacturer’s protocol, with a final reaction volume of 20 μl in each well: 1 μl (about 100 ng) cDNA, 1 μl of each sense and anti-sense primer (10 μM), 10 μl of 2 × SYBR Green Premix and 7 μl ddH_2_O. The PCR reaction was conducted with 95 °C for 60 s, followed by 40 cycles of 95 °C for 10 s, 57 °C for 30 s and 72 °C for 35 s. In order to confirm the specificity of amplification, each reaction was performed with a dissociation curve. The reaction solution without cDNA template was used as negative controls to confirm template-specific amplification. The PCR reaction for each of three biological replicas was implemented according to above-described procedure, and the detection of each gene was performed with three technical replications in an independent sample.

### Determining stability of candidate reference genes expression

Data analyses were performed independently for each of the five groups: developmental stages, normal tissues of adult amphioxus, and the other three groups (i.e. challenged intestine, gill and hepatic caecum with LPS). Average Ct value from three biological replicates was further analyzed according to previous studies^55^. The stability of thirteen RGs was analyzed by five algorithms: geNorm^28^, NormFinder^29^, BestKeeper^30^, deltaCt method^31^ and RefFinder^32^. The geNorm evaluates expression stability of each RG by calculating value (M-value) and excludes candidate one with the highest M-value (less stable) by stepwise cycles. This software also calculates pairwise variation between each RG and the other RGs to determine the optimal number of RGs required for normalization. The NormFinder ranks candidate RGs by calculating their stability value (SV) and standard error among samples in the given group^29^, and the higher expression stability of each gene shows a lower SV. The candidate RGs with a low SV are considered as the reliable ones in BestKeeper^30^. The deltaCt method calculates relative expression levels (REL) of gene pairs between one RG and the other RGs within each sample, and the candidate RGs with the smaller SD value of REL are more stable^31^. Finally, we comprehensively ranked candidates RGs based on the above results from four different statistical applets using a web-based analysis tool RefFinder (http://www.leonxie.com/referencegene.php). The RefFinder examines the stability of candidate RGs by calculating Geometric Mean (GM) values, and RGs with the lower GM values are considered as more stable ones.

### Determination for validation of reference genes selection

To confirm the reliability of the RGs, the relative expression profiles of *EYA* and *HHEX* were determined in thirteen developmental stages and independently normalized with the three most stable RGs [*S20*, *18* *S* and *EF1A*; NF (1–3)], the seven top stable RGs [*S20*, *18* *S*, *EF1A*, *CYC*, *G6PDH*, *UBC* and *RPL3*; NF (1–7)] and the two least stable RGs [*ACTIN* and *RPL13*; NF (12–13)]. Relative quantification of these two target genes was calculated using the 2^−ΔΔCt^ method^56^. Statistical analysis of data was performed by using the IBM SPSS statistics 22 based on LSD test of one-way ANOVA. Products of statistical plots were performed by SigmaPlot 12.0.

## Additional Information

**How to cite this article**: Zhang, Q.-L. *et al.* Selection of reliable reference genes for normalization of quantitative RT-PCR from different developmental stages and tissues in amphioxus. *Sci. Rep.* **6**, 37549; doi: 10.1038/srep37549 (2016).

**Publisher’s note:** Springer Nature remains neutral with regard to jurisdictional claims in published maps and institutional affiliations.

## Supplementary Material

Supplementary Information

## Figures and Tables

**Table 1:** Description of thirteen reference genes in *B. belcheri* for qRT-PCR analysis.

Gene	gene ID	Primer Sequence	Amplicon Size (bp)	PCR Efficiency (E%)/Correlation Coefficient (R^2^)
*18* *S*	M97571.1^#^	F: CCTGAGAAACGGCTACCACA	135	98.60/0.994
		R: ATTTAAAGTGTACCCATTC		
*ACT*	249410 R	F: ATCGTCCGTGACATCAAGGA	178	97.35/0.999
		R: GGAAGGAAGGCTGGAAGAGA		
*CYC*	019450 F	F: CTTCGACATTACCGCTGATGG	120	101.78/0.998
		R: GGAAGTTCTCTGCCGTCTTG		
*EF1A*	210670 F	F: CTGTGCCGTGCTGATTGTA	145	103.89/0.998
		R: GGTGGAGTCCATCTTGTTGAC		
*G6PDH*	065110 R	F: CGGCCAGTGTAAGCGTAAC	181	100.37/0.997
		R: CAGGATGAGTCGTTCGTATGC		
*GAPDH*	215800 F	F: TGACCTGCCGACTGAAGAA	124	96.76/0.999
		R: AAGTCCGAGGACACCACAT		
*HPRT*	238710 R	F: CTTCTTCTCCGACCTGACC	186	93.87/0.998
		R: TCCTCCACAATGAGCACAT		
*L3*	025620 F	F: ACAAGGGTCTGCGTAAGGT	104	101.35/0.990
		R: CTCAGTGCGGTGGTTATAGC		
*L13*	247350 F	F: TGGTGCTGAGGAGTTGAAGA	125	101.65/0.999
		R: ACTGGAACGCCTGGTACTT		
*S20*	107260 F	F: ACTCAGATCCACCGCATCA	112	100.24/0.998
		R: CCTTCACCTGGAGGTTCTTCT		
*TBP*	122820 R	F: TGCCAGAGTGGTACAGAAGT	176	100.04/0.993
		R: ATACGGTAGATCAGGCCAGG		
*TUB*	227130 F	F: CCGTGTGCATGTTGAGTAA	184	100.71/0.996
		R: ACCTCCTCGTAGTCCTTCT		
*UBC*	063240 R	F: TGTCCATCTGCTCACTGCTA	130	99.66/0.995
		R: TGGCGTATCTCTTGGTCCAT		
EYA^*^	223870 R	F: CCAAGCACCACAACCACATATC	306	95.72/0.995
		R: CAGCCAGGTTGAAGATCATCT		
HHEX^*^	128560 F	F: TACCCGCACAGTCTCCTA	106	100.5/0.990
		R: TCTGCGACTCGAACTTCTT		

^#^DNA sequences download directly from Genbank. *Two target genes.

**Table 2:** Stability ranking of the candidate reference genes using five algorithms in different developmental stages of *B. belcheri*.

Gene	RefFinder		geNorm		NormFinder		BestKeeper		deltaCt	
	Rank	GM	Rank	SV	Rank	SV	Rank	SV	Rank	SV
*S20*	1	1.72	3	0.85	2	0.72	1	0.51	2	1.84
*18* *S*	2	2.23	1	0.76	3	0.76	2	0.55	5	1.89
*EF1A*	3	2.74	7	1.49	1	0.64	7	1.71	1	1.82
*CYC*	4	3.11	1	0.76	5	0.84	3	0.63	4	1.88
*G6PDH*	5	4.79	4	0.95	9	1.03	4	0.67	3	1.86
*UBC*	6	5.92	6	1.29	6	0.92	6	1.28	8	2.04
*L3*	7	6.22	8	1.63	4	0.79	9	1.82	6	1.94
*TBP*	8	7.04	5	1.15	10	1.07	5	0.84	10	2.12
*TUB*	9	8.67	9	1.71	7	0.95	10	1.86	7	2.03
*GAPDH*	10	9.41	10	1.76	8	1.02	8	1.74	9	2.10
*HPRT*	11	11.37	11	1.85	12	1.46	11	1.89	12	2.59
*ACT*	12	12.26	12	1.99	11	1.41	12	2.59	11	2.54
*L13*	13	12.93	13	2.03	13	1.71	13	2.87	13	2.84

Parameters of ranking were showed by using GM (geometric mean); SV, stability value. If no additional declarations, these abbreviations indicate the same means as above described in other tables.

**Table 3:** Stability ranking of the candidate reference genes using five algorithms in different normal tissues of *B. belcheri*.

Gene	RefFinder		geNorm		NormFinder		BestKeeper		deltaCt	
	Rank	GM	Rank	SV	Rank	SV	Rank	SV	Rank	SV
*EF1A*	1	1.71	1	0.45	2	0.63	1	0.11	1	2.92
*ACT*	2	1.84	1	0.45	6	1.51	2	0.59	3	3.18
*G6PDH*	3	2.83	3	0.90	3	1.08	3	0.85	2	3.14
*TUB*	4	4.96	6	2.25	7	1.54	5	2.02	4	3.26
*HPRT*	5	5.32	4	1.44	5	1.43	4	1.55	7	3.48
*L13*	6	5.96	7	2.47	4	1.39	7	2.29	5	3.40
*TBP*	7	6.77	5	2.04	1	0.60	8	2.43	6	3.46
*UBC*	8	8.11	9	2.78	9	1.94	6	2.23	9	3.91
*GAPDH*	9	8.64	8	2.64	8	1.80	10	2.45	8	3.86
*18* *S*	10	10.07	11	3.15	11	2.37	9	2.43	10	4.28
*CYC*	11	11.35	10	2.93	10	2.27	11	3.06	11	4.33
*L3*	12	12.04	12	3.43	12	3.16	12	3.70	12	4.76
*S20*	13	12.87	13	3.79	13	3.70	13	3.88	13	5.74

**Table 4:** Stability ranking of the candidate reference genes using five algorithms in intestine of *B. belcheri* challenged with LPS.

Gene	RefFinder		geNorm		NormFinder		BestKeeper		deltaCt	
	Rank	GM	Rank	SV	Rank	SV	Rank	SV	Rank	SV
*S20*	1	2.81	1	0.28	1	0.10	7	1.62	1	1.32
*L13*	2	3.30	1	0.28	2	0.23	5	1.61	2	1.37
*EF1A*	3	3.74	5	0.86	7	0.78	9	1.87	6	1.66
*UBC*	4	4.01	3	0.64	3	0.61	10	2.08	3	1.58
*ACT*	5	5.13	6	0.90	4	0.64	6	1.62	4	1.59
*GAPDH*	6	5.31	4	0.81	6	0.76	11	2.25	7	1.69
*HPRT*	7	5.84	8	1.17	5	0.67	4	1.35	5	1.63
*18* *S*	8	6.33	11	1.53	12	1.32	1	0.18	11	2.16
*G6PDH*	9	6.74	9	1.32	8	0.96	2	0.47	9	1.81
*TBP*	10	7.52	10	1.42	10	1.06	3	0.57	10	1.90
*TUB*	11	7.87	7	1.05	9	0.98	8	1.82	8	1.76
*CYC*	12	12.24	12	1.62	11	1.24	12	2.35	12	2.34
*L3*	13	12.89	13	1.81	13	1.80	13	2.45	13	2.60

**Table 5:** Stability ranking of the candidate reference genes using five algorithms in gill of *B. belcheri* challenged with LPS.

Gene	RefFinder		geNorm		NormFinder		BestKeeper		deltaCt	
	Rank	GM	Rank	SV	Rank	SV	Rank	SV	Rank	SV
*S20*	1	1.21	1	0.51	1	0.30	7	1.04	1	1.40
*EF1A*	2	2.95	3	0.78	2	0.41	8	1.10	3	1.44
*UBC*	3	3.84	1	0.51	3	0.45	10	1.20	2	1.44
*ACT*	4	3.91	5	0.97	4	0.49	2	0.64	5	1.51
*TUB*	5	4.98	4	0.91	6	0.56	6	1.03	4	1.49
*18* *S*	6	5.61	10	1.31	10	0.99	1	0.52	11	1.86
*HPRT*	7	6.05	7	1.11	7	0.84	4	0.94	7	1.72
*L13*	8	6.67	6	1.02	5	0.52	9	1.16	6	1.53
*G6PDH*	9	6.77	9	1.27	9	0.90	3	0.72	8	1.79
*TBP*	10	8.61	8	1.20	11	1.02	5	0.96	10	1.84
*GAPDH*	11	9.85	11	1.38	8	0.90	11	1.67	9	1.81
*CYC*	12	11.83	12	1.59	12	1.68	12	1.96	12	2.71
*L3*	13	12.74	13	1.80	13	1.91	13	2.49	13	2.97

**Table 6:** Stability ranking of the candidate reference genes using five algorithms in hepatic caecum of *B. belcheri* challenged with LPS.

Gene	RefFinder		geNorm		NormFinder		BestKeeper		deltaCt	
	Rank	GM	Rank	SV	Rank	SV	Rank	SV	Rank	SV
*S20*	1	0.94	1	0.76	1	0.41	1	0.42	3	1.40
*L13*	2	1.91	1	0.76	3	0.49	2	0.45	1	1.35
*TBP*	3	3.55	3	0.91	4	0.57	3	0.51	4	1.41
*ACT*	4	3.92	5	1.02	2	0.45	7	0.84	2	1.35
*HPRT*	5	4.82	4	0.93	6	0.62	4	0.56	5	1.45
*TUB*	6	6.11	6	1.06	7	0.70	5	0.69	7	1.52
*EF1A*	7	6.76	7	1.11	5	0.61	9	1.06	6	1.48
*CYC*	8	7.72	8	1.16	8	0.81	6	0.82	8	1.61
*UBC*	9	9.81	9	1.26	9	0.87	11	1.23	9	1.69
*GAPDH*	10	10.04	10	1.33	10	0.97	10	1.21	10	1.79
*G6PDH*	11	10.32	11	1.41	11	1.01	8	0.96	11	1.83
*18* *S*	12	11.91	12	1.46	12	1.01	12	1.26	12	1.85
*L3*	13	12.52	13	1.63	13	1.62	13	2.34	13	2.55
