# Supplementary material for: Selection of reliable reference genes for normalization of quantitative RT-PCR from different developmental stages and tissues in amphioxus
Source: Sci Rep. 2016 Nov 21;6:37549. doi: 10.1038/srep37549 (PMC5116582; doi:10.1038/srep37549)
Supplement: Supplementary Information [file srep37549-s1.pdf]

Supplementary Information

Selection of reliable reference genes for normalization of quantitative RT-PCR from different developmental stages and tissues in amphioxus

Qi-Lin Zhang[1, †], Qian-Hua Zhu[4, †], Xin Liao[1, †], Xiu-Qiang Wang[1], Tao Chen[4], Han-Ting Xu[1], Juan Wang[2], Ming-Long Yuan[2,*] & Jun-Yuan Chen[1, 3*]

[1]Evo-devo Institute, School of Life Sciences, Nanjing University, Nanjing 210008, China.

[2]State Key Laboratory of Grassland Agro-Ecosystems, College of Pastoral Agricultural Science and Technology, Lanzhou University, Lanzhou 730020, China.

[3]Nanjing Institute of Geology and Paleontology, Nanjing 210008, China.

[4]BGI-Shenzhen, Shenzhen 518000, China.

[†]These authors contributed equally to this work.

[*]Correspondence and requests for materials should be addressed to M.L.Y. (yuanml@lzu.edu.cn) or J.Y.C. (chenjy@nju.edu.cn).

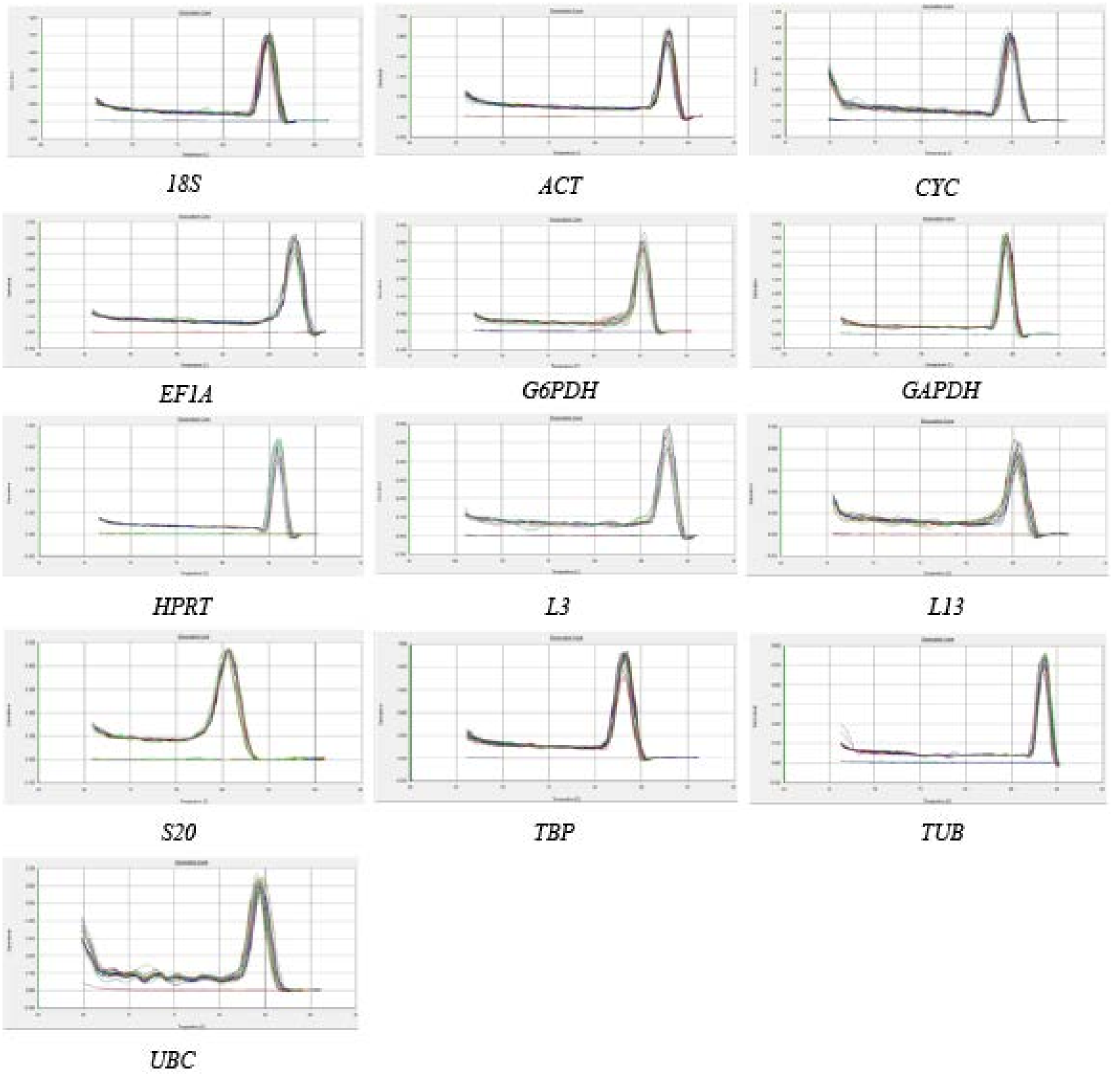

Supplementary Figure S1. Dissociation curves of thirteen candidate reference genes ((*18S*) *18S ribosomal RNA*; (*ACT*) *beta-actin*; (*CYC*) *cyclophilin*; (*EF1A*) *elongation factor 1 alpha*; (*G6PDH*) *glucose 6-phosphate dehydrogenase*; (*GAPDH*) *glyceraldehyde-3-phosphate dehydrogenase*; (*HPRT*) *hypoxanthine-guanine Phosphoribosyltransferase*; (*L3*) *ribosomal protein L3*; (*L13*) *ribosomal protein L13*; (*S20*) *ribosomal protein S20;* (*TBP*) *TATA box binding protein*; (*TUB*) *alpha-tubulin*; (*UBC*) *ubiquitin-conjugating enzyme*) reveal single peaks, which obtained from three technical replicates of different cDNA samples along with no template control.

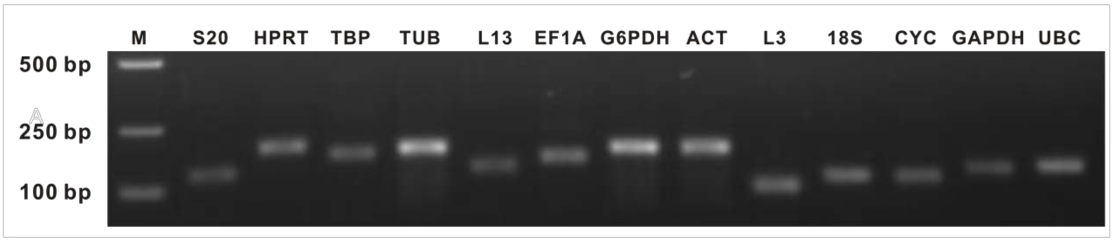

Supplementary Figure S2. 1.5 % agarose gel electrophoresis is exhibiting specific qRT-PCR products with expected size for each candidate reference gene. (M) DNA marker (Takara, DL 2000); (*S20*) *ribosomal protein S20*; (*HPRT*) *hypoxanthine-guanine Phosphoribosyltransferase*; (*TBP*) *TATA box binding protein*; (*TUB*) *alpha-tubulin*; (*L13*) *ribosomal protein L13*; (*EF1A*) *elongation factor 1 alpha*; (*G6PDH*) *glucose 6-phosphate dehydrogenase*; (*ACT*) *beta-actin*; (*L3*) *ribosomal protein L3*; (*18S*) *18S ribosomal RNA*; (*CYC*) *cyclophilin*; (*GAPDH*) *glyceraldehyde-3-phosphate dehydrogenase*; (*UBC*) *ubiquitin-conjugating enzyme*.

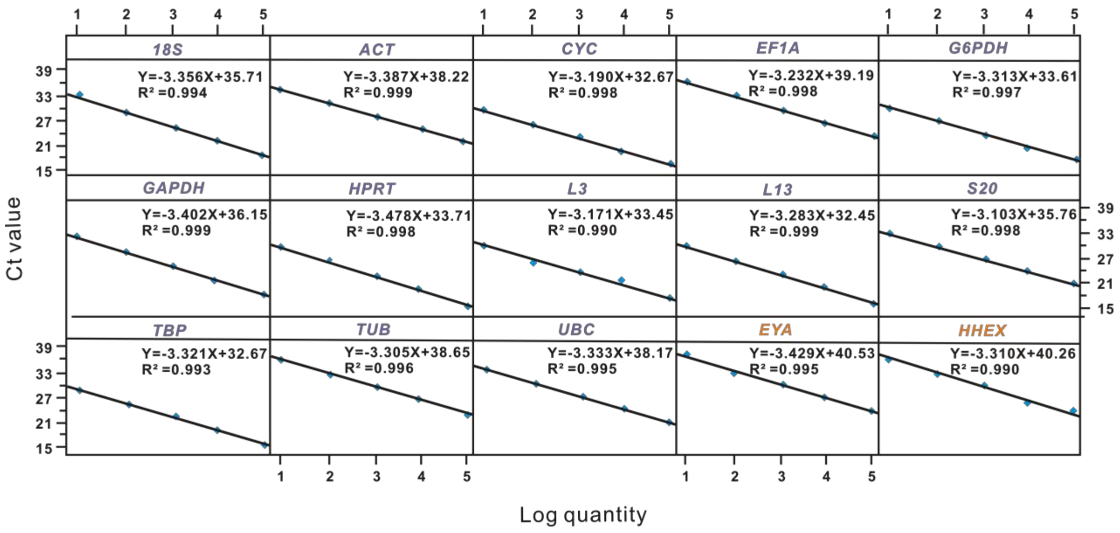

Supplementary Figure S3. Standard curves of thirteen candidate reference genes and two target genes. Line relations and correlation coefficient (R^2) between Ct values and Log quantity were calculated by SigmaPlot 12.0 software.

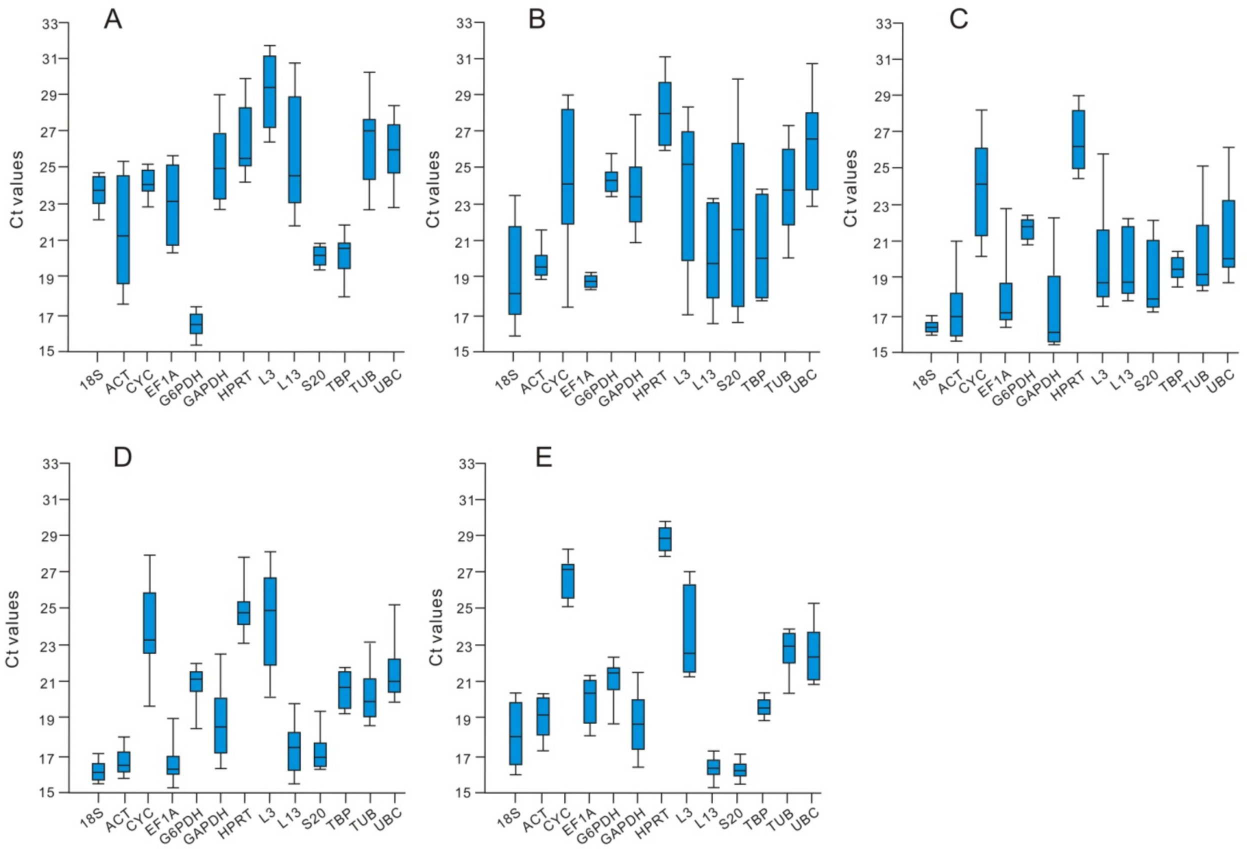

Supplementary Figure S4. Ct values of thirteen candidate housekeeping genes among samples for each developmental condition. Including different developmental stages (A), different normal tissues (B), challenged intestine with LPS (C), challenged gill with LPS (D) and challenged hepatic caecum with LPS (E). The line in the box indicates median. The box describes the 25th and 75th percentiles, and the maximum and minimum values were represented by up and low caps.

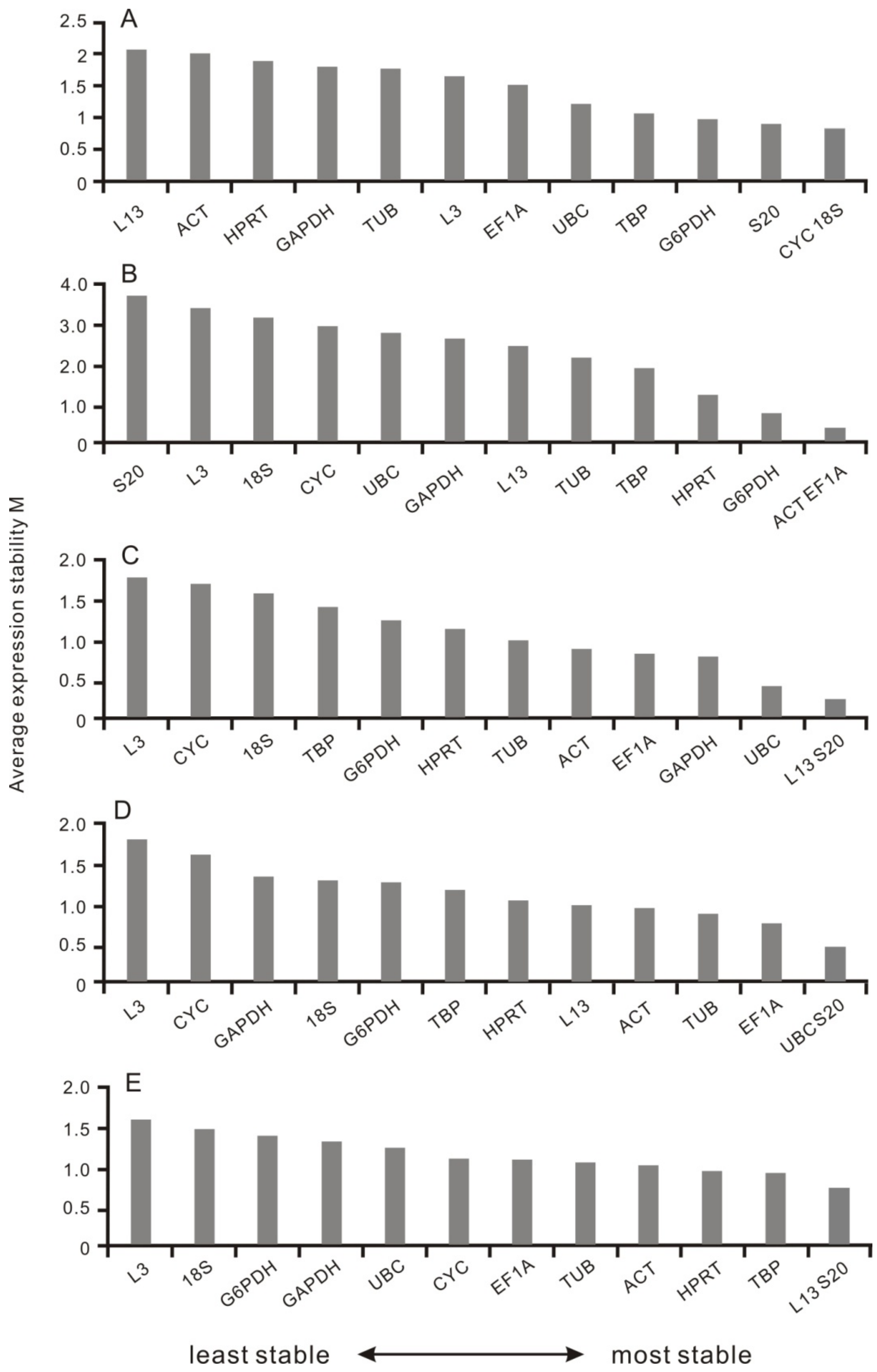

Supplementary Figure S5. geNorm ranking of expression stability of 13 candidate reference genes under five different experimental conditions. Different developmental stage (A), different normal tissues (B), challenged intestine (C), challenged gill (D) and challenged hepatic caecum (E).

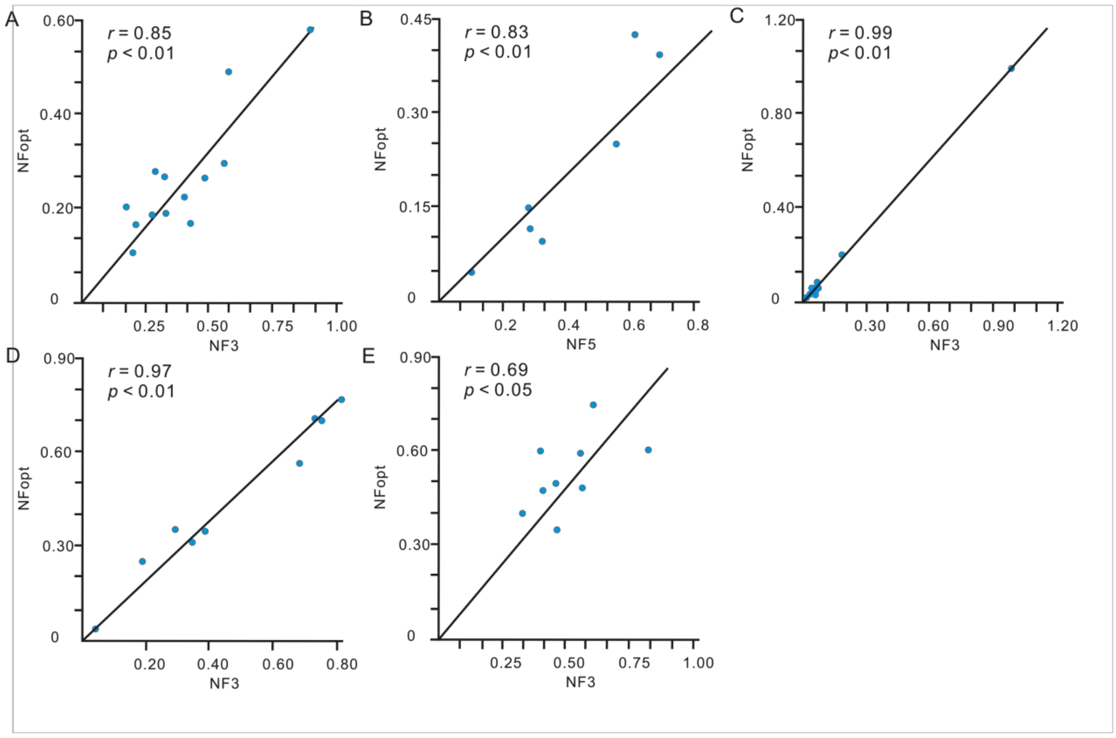

Supplementary Figure S6. Pearson correlations between optimal normalization factors (NF_{opt}) from geNorm software and three or five normalization factor (NF_3 or NF_5) for different developmental stages (A), different normal tissues (B), challenged intestine (C), challenged gill (D) and challenged hepatic caecum (E), which were evaluated by IBM SPSS statistics 22 software.

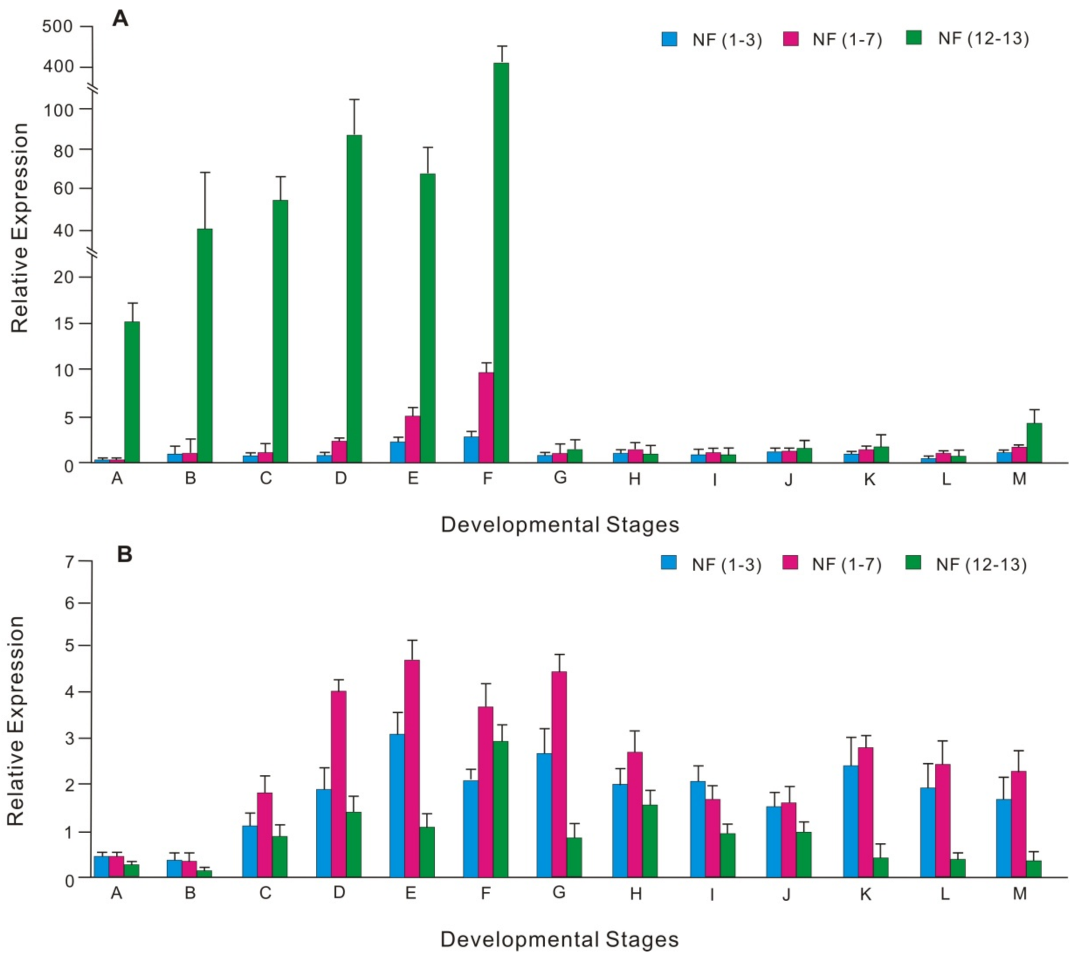

Supplementary Figure S7. Expression profiles of the *EYA* (A) and *HHEX* (B) based on different normalization factors under different developmental stages. A, 8-cell stage; B, morula stage; C, blastula stage; D, middle gastrula stage; E, late gastrula stage; F, neurula stage; G, hatching stage; H,10-somites stage; I, mouse-opening stage; J, 2-gill arch stage; K, two weeks after fertilization ; L, two month after fertilization; M, adult stage. The normalization of *EYA* were performed by using the top three [NF (1-3)], the top seven reference genes [NF (1-7)], and two reference gene with the least stability (NF12-13). Data were exhibited as mean delta Ct values ± their standard deviations.

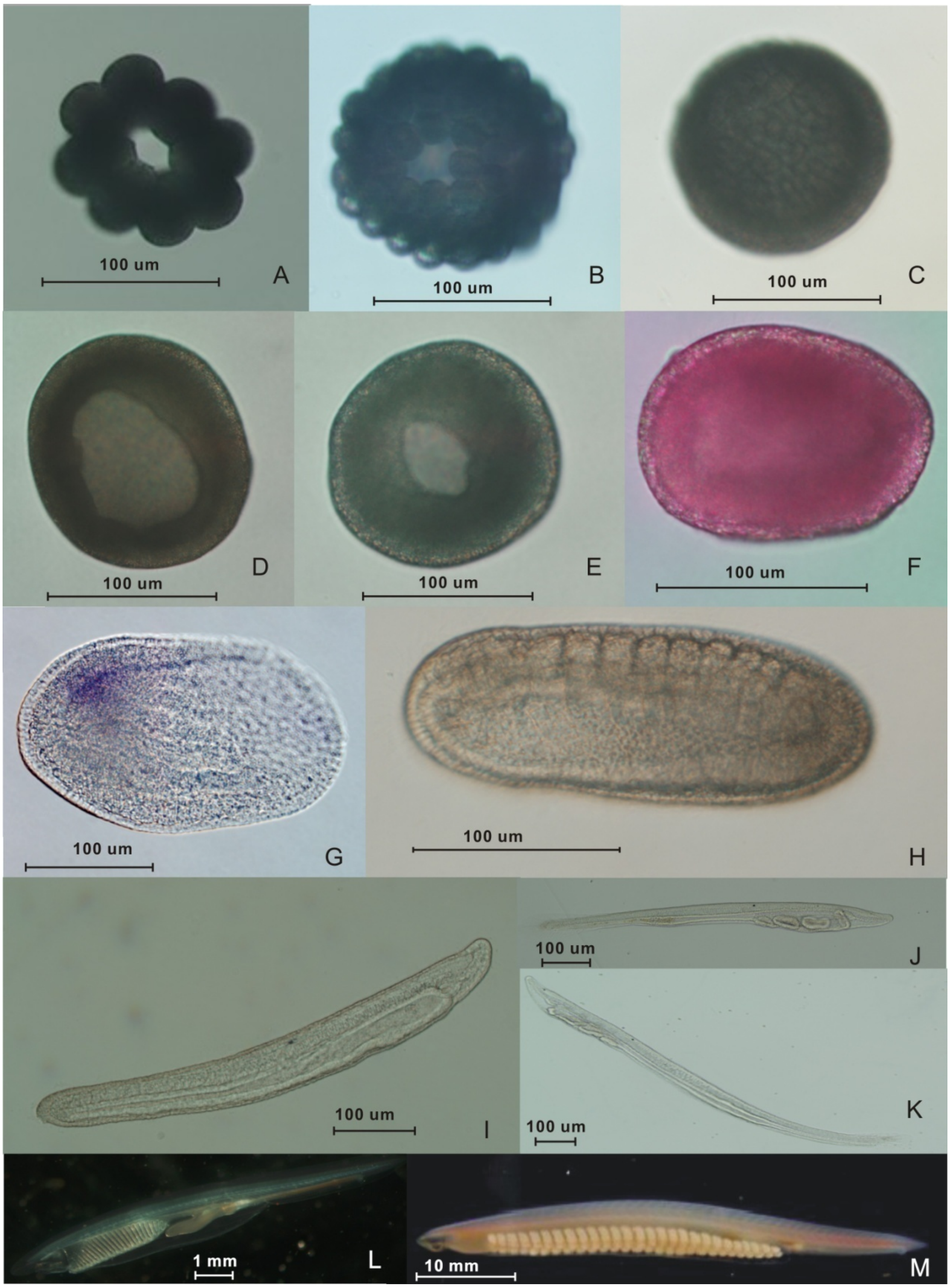

Supplementary Figure S8. Different developmental stages of *B. belcheri*, including 8-cell stage (A), morula (B), blastula (C), middle gastrula stage (D), late gastrula stage (E), neurula (F), hatching stage(G), 10 somites (H), mouse-opening stage (I), 2-gill arch stage (J), two weeks after fertilization (K), two month after fertilization (L), adult stage (M). Photographed by Olympus DP71.

Supplementary Table S1. The mean and standard deviation (SD) of the Ct values under five different experimental conditions.

		18S	*ACT*	*CYC*	*EF1A*	*G6PDH*	*GAPDH*	*HPRT*	*L3*	*L13*	*S20*	*TBP*	*TUB*	*UBC*
Different development stages	Ctmean	23.35	21.44	24.22	23.06	16.48	25.05	26.54	29.02	25.28	20.11	20.16	25.90	25.41
	SD	0.88	2.98	0.82	1.96	0.72	2.31	2.17	2.05	3.30	0.61	1.17	2.31	1.68
Different tissues	Ctmean	18.89	19.83	24.33	18.54	24.29	23.24	28.15	23.85	19.91	21.92	20.73	23.84	26.63
	SD	3.02	0.85	4.08	0.17	0.85	1.07	1.86	4.33	2.75	4.99	2.75	2.58	2.80
Challenged intestine	Ctmean	16.60	17.50	23.88	17.42	21.31	17.21	26.37	20.22	19.47	19.10	19.55	19.94	21.14
	SD	0.27	2.33	2.80	2.53	0.60	2.87	1.64	3.33	1.88	1.95	0.70	2.33	2.53
Challenged gill	Ctmean	15.72	16.27	23.66	16.83	20.84	18.65	24.93	24.67	17.32	16.82	20.65	20.04	21.63
	SD	0.68	0.85	2.63	1.53	0.99	2.20	1.46	3.33	1.47	1.48	1.11	1.41	1.70
Challenged hepatic caecum	Ctmean	18.27	19.21	26.84	20.10	21.23	18.80	28.82	23.84	16.46	15.91	19.48	22.93	22.39
	SD	1.57	1.07	1.04	1.23	1.32	1.60	0.66	2.67	0.67	0.53	0.64	1.00	1.63

See Figure S1 for full names of thirteen reference genes.

Supplementary Table S2. Intra-group variation estimates by NormFinder for five different experimental conditions.

	18S	*ACT*	*CYC*	*EF1A*	*G6PDH*	*GAPDH*	*HPRT*	*L3*	*L13*	*S20*	*TBP*	*TUB*	*UBC*
Different development stages	0.183	0.301	0.196	0.162	0.228	0.227	0.310	0.187	0.359	0.175	0.236	0.215	0.209
Different tissues	0.723	0.501	0.697	0.347	0.405	0.573	0.484	0.940	0.474	1.089	0.346	0.508	0.610
Challenged intestine	0.343	0.192	0.326	0.220	0.260	0.216	0.197	0.458	0.148	0.239	0.283	0.265	0.185
Challenged gill	0.266	0.164	0.430	0.152	0.247	0.246	0.233	0.484	0.169	0.144	0.274	0.176	0.158
Challenged hepatic caecum	0.267	0.147	0.220	0.178	0.266	0.257	0.180	0.412	0.155	0.141	0.169	0.197	0.233

Supplementary Table S3. Pairwise comparison of candidate reference genes for each experimental condition*.

	Gene		Pair1	Pair2	Pair3	Pair4	Pair5	Pair6	Pair7	Pair8	Pair9	Pair10	Pair11	Pair12	Avg SD
Different	*EF1A*	Mean	1.95	-1.64	2.75	-2.89	2.28	0.19	3.56	1.05	-2.97	5.92	2.30	-6.68	
developmental stages		SD	1.83	1.55	1.20	2.44	2.00	1.61	2.76	1.94	1.76	0.77	1.77	2.26	1.82
	S20	Mean	4.92	1.32	5.71	0.07	5.24	2.97	3.16	6.52	4.02	8.89	5.27	-3.71	
		SD	2.29	2.70	2.28	1.42	1.83	1.76	0.87	2.19	0.88	1.76	3.02	1.08	1.84
	G6PDH	Mean	1.76	-1.83	2.56	-3.08	2.09	-0.19	3.37	0.86	-3.16	5.73	2.11	-6.87	
		SD	2.40	2.73	2.10	1.69	1.59	1.61	2.55	0.80	0.87	1.65	2.98	1.36	1.86
	CYC	Mean	0.90	-2.69	1.69	-3.95	1.22	-1.05	-0.86	2.50	-4.02	4.87	1.25	-7.73	
		SD	2.11	3.05	2.34	1.31	1.23	1.94	0.80	2.27	0.88	2.09	3.32	1.22	1.88
	18S	Mean	8.63	5.03	9.42	3.78	8.95	6.68	6.87	10.23	7.73	3.71	12.60	8.98	
		SD	2.36	2.31	2.40	0.94	1.81	2.26	1.36	2.20	1.22	1.08	2.33	2.43	1.89
	L3	Mean	-3.97	-7.56	-3.17	-8.81	-3.64	-5.92	-5.73	-2.36	-4.87	-8.89	-3.62	-12.60	
		SD	2.30	1.60	1.57	2.53	2.25	0.77	1.65	2.90	2.09	1.76	1.55	2.33	1.94
	TUB	Mean	-0.84	-4.46	-5.74	-0.49	-2.84	-2.55	0.64	-1.68	-5.79	3.12	-0.62	-9.42	
		SD	1.71	1.82	2.40	1.98	1.20	2.01	2.88	2.25	2.20	1.51	2.12	2.29	2.03
	UBC	Mean	-0.32	-3.92	0.47	-5.17	-2.28	-2.09	1.28	-1.22	-5.24	3.64	0.02	-8.95	
		SD	1.53	3.08	2.07	1.60	2.00	1.59	2.15	1.23	1.83	2.25	3.29	1.81	2.04
	GAPDH	Mean	-3.62	0.84	-4.89	0.36	-1.99	-1.71	1.48	-0.83	-4.94	3.97	0.22	-8.57	
		SD	2.33	1.71	2.08	1.47	1.76	2.31	2.01	2.03	2.20	2.20	2.90	2.27	2.10
	TBP	Mean	4.85	1.25	5.64	5.17	2.89	3.08	6.45	3.95	-0.07	8.81	5.19	-3.78	
		SD	2.16	3.32	2.48	1.60	2.44	1.69	1.98	1.31	1.42	2.53	3.60	0.94	2.12
	ACT	Mean	3.62	4.46	-1.27	3.98	1.63	1.91	5.10	2.79	-1.33	7.59	0.22	-4.95	
		SD	2.33	1.82	3.18	2.96	1.48	2.63	2.98	2.94	2.59	1.53	2.90	3.14	2.54
	HPRT	Mean	-1.60	-5.20	-0.81	-6.45	-1.28	-3.56	-3.37	-2.50	-6.52	2.36	-1.26	-10.23	
		SD	2.05	3.09	2.94	1.98	2.15	2.76	2.55	2.27	2.19	2.90	3.96	2.20	2.59

	L13	Mean	-0.35	-3.94	0.45	-5.19	-0.02	-2.30	-2.11	1.26	-1.25	-5.27	3.62	-8.98	
		SD	3.00	1.91	2.11	3.60	3.29	1.77	2.98	3.96	3.32	3.02	1.55	3.53	2.84
Different normal tissues	*EF1A*	Mean	4.70	1.28	5.30	2.19	8.09	5.75	9.61	5.78	3.38	5.31	1.36	0.34	
		SD	3.45	0.83	2.63	2.79	2.89	1.06	1.86	4.12	5.06	4.37	2.87	3.06	2.92
	G6PDH	Mean	-1.05	-4.47	-0.45	-3.56	2.33	-5.75	3.86	0.03	-2.37	-0.44	-4.39	-5.41	
		SD	3.60	1.29	3.23	3.49	3.22	1.06	2.60	4.73	4.15	4.95	3.22	2.21	3.14
	ACT	Mean	3.42	4.31	1.44	6.05	-0.31	3.58	7.59	4.66	3.02	3.41	0.56	-0.24	
		SD	3.23	2.61	2.62	4.14	1.86	3.12	2.63	3.78	4.17	4.13	3.09	2.78	3.18
	TUB	Mean	-0.60	-4.02	-3.11	2.79	-5.30	0.45	4.31	0.48	-1.92	0.01	-3.93	-4.96	
		SD	2.19	2.50	1.14	3.68	2.63	3.23	3.05	2.65	6.55	3.68	2.90	4.93	3.26
	L3	Mean	3.34	-0.08	3.93	0.83	6.72	-1.36	4.39	8.24	4.42	2.02	3.94	-1.02	
		SD	3.41	2.48	2.90	3.24	3.43	2.87	3.22	2.77	3.07	5.57	3.33	4.49	3.40
	TBP	Mean	2.51	-0.91	3.11	5.90	-2.19	3.56	7.42	3.59	1.19	3.12	-0.83	-1.85	
		SD	2.11	2.79	1.14	3.05	2.79	3.49	3.37	3.28	6.81	4.45	3.24	4.99	3.46
	HPRT	Mean	-4.91	-8.32	-4.31	-7.42	-1.52	-9.61	-3.86	-3.82	-6.23	-4.30	-8.24	-9.26	
		SD	4.50	1.80	3.05	3.37	3.94	1.86	2.60	3.30	6.31	3.61	2.77	4.63	3.48
	GAPDH	Mean	-3.42	0.60	-2.51	3.38	-4.70	1.05	4.91	1.08	-1.32	0.61	-3.34	-4.36	
		SD	3.53	2.19	2.11	3.19	3.45	3.60	4.50	4.46	5.71	5.53	3.68	4.39	3.86
	UBC	Mean	-3.38	-6.80	-2.79	-5.90	-8.09	-2.33	1.52	-2.30	-4.70	-2.78	-6.72	-7.74	
		SD	3.19	3.22	3.68	3.05	2.89	3.22	3.94	5.45	5.34	6.03	3.43	3.55	3.91
	18S	Mean	4.36	0.94	4.96	1.85	7.74	-0.34	5.41	9.26	5.44	3.04	4.96	1.02	
		SD	4.39	3.32	4.93	4.99	3.55	3.06	2.21	4.63	6.66	2.42	6.69	4.49	4.28
	CYC	Mean	-1.08	-4.50	-0.48	-3.59	2.30	-5.78	-0.03	3.82	-2.40	-0.48	-4.42	-5.44	
		SD	4.46	3.62	2.65	3.28	5.45	4.12	4.73	3.30	8.00	2.60	3.07	6.66	4.33
	L3	Mean	-0.61	-4.02	-0.01	-3.12	2.78	-5.31	0.44	4.30	0.48	-1.93	-3.94	-4.96	

		SD	5.53	3.99	3.68	4.45	6.03	4.37	4.95	3.61	2.60	7.83	3.33	6.69	4.76
	S20	Mean	1.32	-2.10	1.92	-1.19	4.70	-3.38	2.37	6.23	2.40	1.93	-2.02	-3.04	
		SD	5.71	5.17	6.55	6.81	5.34	5.06	4.15	6.31	8.00	7.83	5.57	2.42	5.74
Challenged insteine	*S20*	Mean	-1.89	-1.60	0.84	0.45	2.04	-1.68	2.21	7.27	4.78	1.12	0.37	-2.50	
with LPS		SD	1.16	1.09	1.29	1.63	0.80	1.04	1.57	1.13	1.85	1.99	0.29	1.99	1.32
	L13	Mean	-2.26	-1.96	0.47	0.08	1.67	-2.04	1.84	6.90	4.42	-0.37	0.76	-2.87	
		SD	1.35	1.21	1.51	1.55	0.90	1.21	1.53	1.17	1.70	0.29	2.07	1.96	1.37
	UBC	Mean	-3.93	-3.39	-1.37	-1.59	-3.71	0.17	5.23	2.75	-2.04	-0.91	-1.67	-4.54	
		SD	0.69	1.38	1.94	2.10	0.74	2.14	1.45	1.83	0.80	2.34	0.90	2.59	1.58
	ACT	Mean	-0.30	2.44	2.05	3.63	-0.08	3.81	8.87	6.38	1.60	2.72	1.96	-0.90	
		SD	1.01	1.22	1.99	1.09	0.55	2.02	1.68	2.16	1.09	2.68	1.21	2.38	1.59
	HPRT	Mean	-9.16	-8.87	-6.43	-6.82	-5.23	-8.95	-5.06	-2.49	-7.27	-6.15	-6.90	-9.77	
		SD	1.72	1.68	1.44	1.39	1.45	1.64	1.20	2.55	1.13	2.61	1.17	1.60	1.63
	EF1A	Mean	-0.14	0.08	2.52	2.13	3.71	4.66	7.58	6.51	3.12	2.73	2.01	0.36	
		SD	0.58	0.55	1.26	2.16	0.74	1.01	3.95	1.63	2.78	2.57	1.15	1.59	1.66
	GAPDH	Mean	0.30	2.73	2.34	3.93	0.22	4.11	9.16	6.68	1.89	3.02	2.26	-0.61	
		SD	1.01	1.27	2.52	0.69	0.58	2.46	1.72	2.28	1.15	2.32	1.35	2.89	1.69
	TUB	Mean	-2.73	-2.44	-0.39	1.20	-2.52	1.37	6.43	3.94	-0.84	0.28	-0.47	-3.34	
		SD	1.27	1.22	2.25	1.57	1.26	1.94	1.44	2.90	1.29	2.26	1.51	2.25	1.76
	G6PDH	Mean	-4.10	-3.81	-1.37	-1.76	-0.17	-3.89	5.06	2.57	-2.21	-1.09	-1.84	-4.71	
		SD	2.46	2.02	1.94	0.69	2.14	2.15	1.20	2.51	1.57	2.93	1.53	0.58	1.81
	TBP	Mean	-2.34	-2.05	0.39	1.59	-2.13	1.76	6.82	4.33	-0.45	0.67	-0.08	-2.95	
		SD	2.53	1.99	2.25	2.10	2.16	0.69	1.39	2.37	1.63	3.25	1.55	0.89	1.90
	18S	Mean	0.61	0.90	3.34	2.95	4.54	0.82	4.71	9.77	7.28	2.50	3.62	2.87	
		SD	2.89	2.38	2.25	0.89	2.59	2.56	0.58	1.60	2.95	1.99	3.30	1.96	2.16

	CYC	Mean	-6.68	-6.38	-3.94	-4.33	-2.75	-6.46	-2.57	2.49	-4.78	-3.66	-4.42	-7.28	
		SD	2.29	2.16	2.90	2.37	1.83	2.05	2.51	2.55	1.85	2.87	1.70	2.95	2.34
	L3	Mean	-3.02	-2.72	-0.28	-0.67	0.91	-2.80	1.09	6.15	3.66	-1.12	-0.76	-3.62	
		SD	2.32	2.68	2.26	3.25	2.34	2.56	2.93	2.61	2.87	1.99	2.07	3.30	2.60
Challenged gill with LPS	*S20*	Mean	1.83	-0.55	3.22	3.83	4.81	0.01	4.02	8.11	6.84	7.85	0.50	-1.10	
		SD	1.27	1.08	0.91	1.53	0.51	1.01	1.60	1.24	2.44	2.42	1.25	1.58	1.40
	EF1A	Mean	1.82	-0.56	3.21	3.82	4.79	4.01	8.10	6.83	-0.01	7.84	0.49	-1.11	
		SD	0.98	0.97	1.11	1.80	0.82	1.57	1.52	2.46	1.01	2.52	0.69	1.78	1.44
	UBC	Mean	-2.97	-5.35	-1.58	-0.97	-4.79	-0.78	3.31	2.03	-4.81	3.04	-4.30	-5.90	
		SD	0.84	1.12	1.10	1.79	0.82	1.71	1.56	2.57	0.51	2.16	1.21	1.83	1.44
	TUB	Mean	-1.39	-3.77	0.61	1.58	-3.21	0.80	4.89	3.62	-3.22	4.63	-2.72	-4.32	
		SD	1.73	1.05	0.96	1.10	1.11	1.62	0.74	2.84	0.91	2.97	1.37	1.44	1.49
	ACT	Mean	2.38	3.77	4.38	5.35	0.56	4.57	8.66	7.39	0.55	8.40	1.05	-0.55	
		SD	1.57	1.05	1.19	1.12	0.97	0.92	1.34	2.75	1.98	3.09	1.10	1.07	1.51
	L13	Mean	1.33	-1.05	2.72	3.33	4.30	-0.49	3.52	7.61	6.34	-0.50	7.35	-1.60	
		SD	1.24	1.10	1.37	1.95	1.21	0.69	1.24	1.65	2.07	1.25	2.71	1.84	1.53
	HPRT	Mean	-6.28	-8.66	-4.89	-4.28	-3.31	-8.10	-4.09	-1.27	-8.11	-0.26	-7.61	-9.21	
		SD	2.13	1.34	0.74	1.02	1.56	1.52	1.76	2.75	1.24	3.35	1.65	1.58	1.72
	G6PDH	Mean	-2.19	-4.57	-0.80	-0.19	0.78	-4.01	4.09	2.82	-4.02	3.83	-3.52	-5.12	
		SD	2.00	0.92	1.62	1.60	1.71	1.57	1.76	2.60	1.60	3.51	1.24	1.39	1.79
	GAPDH	Mean	-2.38	1.39	2.00	2.97	-1.82	2.19	6.28	5.01	-1.83	6.02	-1.33	-2.93	
		SD	1.57	1.73	2.47	0.84	0.98	2.00	2.13	2.47	1.27	2.52	1.24	2.48	1.81
	TBP	Mean	-2.00	-4.38	-0.61	0.97	-3.82	0.19	4.28	3.01	-3.83	4.02	-3.33	-4.93	
		SD	2.47	1.19	0.96	1.79	1.80	1.60	1.02	3.26	1.53	3.58	1.95	0.92	1.84
	18S	Mean	2.93	0.55	4.32	4.93	5.90	1.11	5.12	9.21	7.94	1.10	8.94	1.60	

		SD	2.48	1.07	1.44	0.92	1.83	1.78	1.39	1.58	3.09	1.58	3.28	1.84	1.86
	CYC	Mean	-5.01	-7.39	-3.62	-3.01	-2.03	-6.83	-2.82	1.27	-6.84	1.01	-6.34	-7.94	
		SD	2.47	2.75	2.84	3.26	2.57	2.46	2.60	2.75	2.44	3.28	2.07	3.09	2.71
	L3	Mean	-6.02	-8.40	-4.63	-4.02	-3.04	-7.84	-3.83	0.26	-1.01	-7.85	-7.35	-8.94	
		SD	2.52	3.09	2.97	3.58	2.37	2.52	3.51	3.35	3.28	2.42	2.71	3.28	2.97
Challenged hepatic caecum with LPS	*L13*	Mean	2.34	2.76	6.48	3.03	5.93	3.64	4.78	12.37	10.38	-0.55	7.39	1.81	
		SD	1.32	1.11	1.30	0.95	1.23	1.37	1.86	1.09	0.88	0.76	2.59	1.73	1.35
	ACT	Mean	-0.41	3.72	0.27	3.17	0.89	2.02	9.61	7.63	-3.30	4.63	-2.76	-0.95	
		SD	1.86	0.94	0.96	1.45	0.73	1.80	1.34	1.41	1.20	1.99	1.11	1.47	1.35
	S20	Mean	2.89	3.30	7.03	-4.36	6.48	4.19	5.32	12.91	10.93	7.93	0.55	2.36	
		SD	1.46	1.20	1.27	2.11	1.52	1.33	1.27	0.88	1.01	2.41	0.76	1.54	1.40
	TBP	Mean	-0.69	-0.27	3.45	2.90	0.61	1.75	9.34	7.36	-3.57	4.36	-3.03	-1.22	
		SD	1.86	0.96	0.86	1.86	1.34	1.49	0.89	1.56	0.98	2.48	0.95	1.65	1.41
	HPRT	Mean	-10.02	-9.61	-5.89	-9.34	-6.44	-8.72	-7.59	-1.98	-12.91	-4.98	-12.37	-10.56	
		SD	1.67	1.34	1.35	0.89	1.84	1.37	1.22	1.47	0.88	2.78	1.09	1.47	1.45
	EF1A	Mean	-1.30	-0.89	2.84	-1.75	2.29	1.13	8.72	6.74	-4.19	3.75	-3.64	-1.83	
		SD	1.92	0.73	1.01	1.49	1.56	1.63	1.37	1.46	1.33	2.26	1.37	1.66	1.48
	TUB	Mean	-4.14	-3.72	-3.45	-0.55	-2.84	-1.70	5.89	3.91	-7.03	0.91	-6.48	-4.67	
		SD	2.06	0.94	0.86	1.95	1.01	1.43	1.35	1.69	1.27	2.46	1.30	1.89	1.52
	CYC	Mean	-8.04	-7.63	-3.91	-7.36	-4.45	-6.74	-5.61	1.98	-10.93	-3.00	-10.38	-8.57	
		SD	1.67	1.41	1.69	1.56	1.35	1.46	2.07	1.47	1.01	2.91	0.88	1.82	1.61
	UBC	Mean	-3.59	-3.17	0.55	-2.90	-2.29	-1.15	6.44	4.45	-6.48	1.46	-5.93	-4.12	
		SD	0.90	1.45	1.95	1.86	1.56	2.33	1.84	1.35	1.52	2.44	1.23	1.78	1.69
	GAPDH	Mean	0.41	4.14	0.69	3.59	1.30	2.43	10.02	8.04	-2.89	5.05	-2.34	-0.53	
		SD	1.86	2.06	1.86	0.90	1.92	2.04	1.67	1.67	1.46	2.72	1.32	1.99	1.79

G6PDH	Mean	-2.43	-2.02	1.70	-1.75	1.15	-1.13	7.59	5.61	-5.32	2.61	-4.78	-2.96	
	SD	2.04	1.80	1.43	1.49	2.33	1.63	1.22	2.07	1.27	2.71	1.86	2.09	1.83
18S	Mean	0.53	0.95	4.67	1.22	4.12	1.83	2.96	10.56	8.57	-2.36	5.58	1.81	
	SD	1.99	1.47	1.89	1.65	1.78	1.66	2.09	1.47	1.82	1.74	2.90	1.73	1.85
L3	Mean	-5.05	-4.63	-0.91	-4.36	-1.46	-3.75	-2.61	4.98	3.00	-7.93	-7.39	-5.58	
	SD	2.72	1.99	2.46	2.48	2.44	2.26	2.71	2.78	2.91	2.41	2.59	2.90	2.55

*****deltaCt method was used for the data analysis.
